# Epidemiology and Clinical Characteristics of Pediatric Drug-Resistant Tuberculosis in Chongqing, China

**DOI:** 10.1371/journal.pone.0151303

**Published:** 2016-03-09

**Authors:** Qian Guo, Yun Pan, Zhenhua Yang, Ruixi Liu, Linlin Xing, Zhe Peng, Chaomin Zhu

**Affiliations:** 1 Department of Infectious Diseases, Children’s Hospital of Chongqing Medical University, Chongqing, China; 2 Key Laboratory of Child Development and Disorders, Ministry of Education, Chongqing, China; 3 Key Laboratory of Pediatrics in Chongqing, Chongqing, China; 4 Epidemiology Department, School of Public Health, University of Michigan, Ann Arbor, MI, United States of America; University College London, UNITED KINGDOM

## Abstract

To gain insight into the epidemiology of childhood drug resistant tuberculosis (DR-TB) in China that has the second largest burden of TB and the largest number of multidrug resistant (MDR) TB cases in the world, we performed the cross-sectional study to investigate drug resistance of four first-line anti-TB drugs (isoniazid, rifampicin, streptomycin and ethambutol) using *Mycobacterium tuberculosis* isolates from 196 culture-confirmed pediatric TB cases diagnosed in the Children’s Hospital of Chongqing Medical University, China during 2008–2013. Univariate and multivariate logistic regression analyses were performed to assess the associations between patient demographic and clinical characteristics and DR-and MDR-TB, respectively. Twenty-eight percent (56/196) of the study patients exhibited resistance to at least one of the four first-line anti-TB drugs tested. MDR was found in 4.6% (9/196) of the study patients. More than half (5/9, 55.6%) of the MDR cases were from a single county of Chongqing. A significant association was found between being acid-fast bacilli-smear negative and DR-TB (adjusted OR, 2.33; 95% CI, 1.13–4.80) and between having concurrent thoracic-extrathoracic involvement and MDR-TB (adjusted OR, 9.49; 95% CI, 1.05–85.92), respectively. The findings of this study indicate that the rate of DR is high among pediatric TB patients in Chongqing and suggest an urgent need for studies to identify MDR transmission hotspots in Chongqing, thereby contributing to the control DR- and MDR-TB epidemics in China. The study also generates new insight into the pathogenesis of DR and MDR *M*. *tuberculosis* strains and highlights the importance of studying childhood TB to the goal of global TB control.

## Introduction

Tuberculosis (TB) is an ancient disease, yet it has remained a leading infectious killer of the world’s population up to current time. In 2013 alone, TB caused illness in nine million people and 1.5 million deaths globally [1]. The emergence and spread of drug-resistant (DR) TB, especially multidrug-resistant (MDR) and extensive-drug resistant TB, poses a major challenge to global TB control. The World Health Organization (WHO) estimates that that 3.5% of the new cases and 20.5% of previously treated cases had MDR-TB in 2013 [1]. While it is estimated that childhood TB constitutes up to 10–15% of the global TB caseload [2], investigation of DR-TB in children is limited, largely due to the well-known difficulties to isolate *M*. *tuberculosis* from pediatric specimens.

China, where the current study was carried out, ranks second among the 22 high TB-burden countries and has the world’s largest number of patients with MDR-TB [3]. Yet, the magnitude and clinical characteristics of DR-TB in Chinese children remains largely unknown. The last national TB survey that included childhood TB was conducted in 2000, however, only very limited data on pediatric DR-TB were collected in that survey [4]. More recently Jiao and colleagues characterized DR profiles of 100 *M*. *tuberculosis* isolates from pediatric cases diagnosed in different regions of China [5]. They found an alarmingly high prevalence of pediatric DR-TB (55%, 55/100) and MDR-TB (22%, 22/100) in China. But, their study only included 22 isolates from the West China, where the highest incidence of childhood TB was reported [6]. The actual situation of DR and MDR among pediatric TB cases in the West China remains to be assessed. Furthermore, because pediatric TB is a direct consequence of adult TB, it is a good marker of current transmission occurring in the community. A better understanding of the epidemiology of DR-TB in children in China can shed light on the performance of TB control programs in the country, while informing the development of better strategies and tools for pediatric TB control in China, thereby contributing to the global TB control given the important position of China in the global TB epidemics.

Another important question in DR-TB research is related to the biology of DR *M*. *tuberculosis* strains. It has been proposed by some researchers that DR strains, and especially MDR *M*. *tuberculosis* strains, had a decreased ability to survive and to reproduce *in vitro* and *vivo* [7–10]. A recent study using a murine model of aerosol *M*. *tuberculosis* infection showed that DR isolates harbored a reduced virulence and were less likely to disseminate to peripheral sites from lungs, compared to drug-sensitive isolates [8]. In contrast, an epidemiological study of pediatric DR-TB conducted in Madrid, Spain showed an association between MDR and extrapulmonary TB [11]. The observed association, therefore, remains to be further assessed in other populations to generalize this finding and confirm its important implication for DR-TB pathogenesis. Because pediatric TB affects extrathoracic organs and tissues much more frequently than adult TB does [12], a clinically well-characterized pediatric TB isolate collection provides an excellent opportunity to assess the ability of DR *M*. *tuberculosis* strains to disseminate in the infected host using an epidemiological approach.

To fill in those knowledge gaps described above, we conducted the present retrospective study in Chongqing, the only West China municipality that was under the direct administration of the central government of China. Chongqing has a total population of 33,752,000, including the 21 districts, 13 counties, and 4 autonomous regions under its jurisdiction. The study used *M*. *tuberculosis* isolates and patient data available at the Children’s Hospital of Chongqing Medical University (CHCMU), China, which diagnoses and treats the majority of childhood TB cases in the Chongqing area.

## Materials and Methods

### Study sample and data collection

The study sample included 196 mycobacterial culture-confirmed pediatric TB cases diagnosed at CHCMU during February 1, 2008—December 31, 2013. During this time period, CHCMU diagnosed and treated 2,475 TB patients. Mycobacterial culture was performed for all the patients who were suspected to have TB and based on clinical symptoms and positive tuberculin PPD skin test and for whom biological specimens were successfully obtained. Of these 2,475 patients, 360 were culture-positive and the remaining 2,115 were clinical cases. The TB diagnosis followed the pediatric TB diagnosis guidelines of WHO [13]. The study sample included all available isolates of culture-confirmed cases (n = 196). The other 164 culture-confirmed cases were excluded from the study due to loss of viability of the frozen culture of these isolates. Of the 196 cases, 179 cases were identified to be infected with *M*. *tuberculosis* and the remaining 17 cases were infected with *Mycobacterium bovis* based on the para-nitrobenzoic acid/thiophene-2-carboxylic acid hydrazide test [14]. Demographic (sex, age, and residence) and clinical (acid-fast bacilli [AFB] smear and disease site) data of the study patients were obtained from medical records when they were involved in the study. Written informed consent was obtained from the legal guardians of the study’s pediatric patients. Only de-identified data were used in the data analysis. The study protocol was approved by the Medical Ethics Committee of Children’s Hospital, Chongqing Medical University.

### *M*. *tuberculosis* complex isolates

To determine the degree of DR among TB patients at CHCMU, at least one isolate was obtained from each of the 196 study patients. To examine whether or not isolates from different anatomic sites of an individual patient represent different strains, we obtained 14 additional isolates from different disease sites of 14 of the 196 study patients and investigated the consistency of DR patterns between isolates obtained from different anatomic sites of these 14 patients. The inclusion of these additional isolates from different sites also serves as a validation for that DR pattern of an isolate derived from a single disease site can represent isolates from other sites in a patient having disseminated TB, when isolates from some anatomic sites cannot be isolated.

### Drug susceptibility testing

Drug susceptibility testing (DST) was conducted using proportion method as previously described [3]. Four first-line anti-TB drugs that had been routinely used in China to date, including isoniazid (INH), rifampin (RIF), streptomycin (STR) and ethambutol (EMB), were tested using the following concentrations in the medium: 0.2 μg/ml for INH, 40.0 μg/ml for RIF, 4.0 μg/ml for STR, and 2.0 μg/ml for EMB. Isolate with growth proportion for more than 1% compared to the control was considered to be resistant to a certain anti-TB drug.

### Patient classification and statistical analysis

We calculated the proportions of the study isolates that were resistant to any of the four first-line anti-TB drugs and that were MDR, respectively. Cases whose *M*. *tuberculosis* isolates were resistant to any single drugs or drug combinations were defined as having DR-TB, and the remaining cases were defined as pansensitive cases. The DR group was further divided into MDR and non-MDR DR groups, based on the MDR definition (resistance to at least INH and RIF). To identify risk factors and predictors for having pediatric DR-TB, we compared the frequency distribution of demographic and clinical characteristics between pansensitve and DR groups and between pansensitve and MDR groups, respectively by Chi-square test, or Fisher's exact test, as appropriate. To describe the strength and significance of the associations of demographic and clinical characteristics with having DR or MDR-TB, we obtained crude and adjusted odds ratios (OR) and 95% confidence interval (CI) using univariate and multivariate logistic regression analyses. Motivated by the ongoing debate on the fitness cost of DR and MDR strains, we assessed the association between extrathoracic involvement and DR- and MDR-TB to gain insight to the ability of DR and MDR *M*. *tuberculosis* strains to disseminate within the infected human body. We classified the study subjects into exclusive thoracic, exclusive extrathoracic, and concurrent thoracic-extrathoracic, using definitions described previously [15–17]. P value less than 0.05 was considered to be significant. All statistical analyses were performed using SPSS software, version 17.0.

## Results

### Study patient characteristics

**Fig 1:**
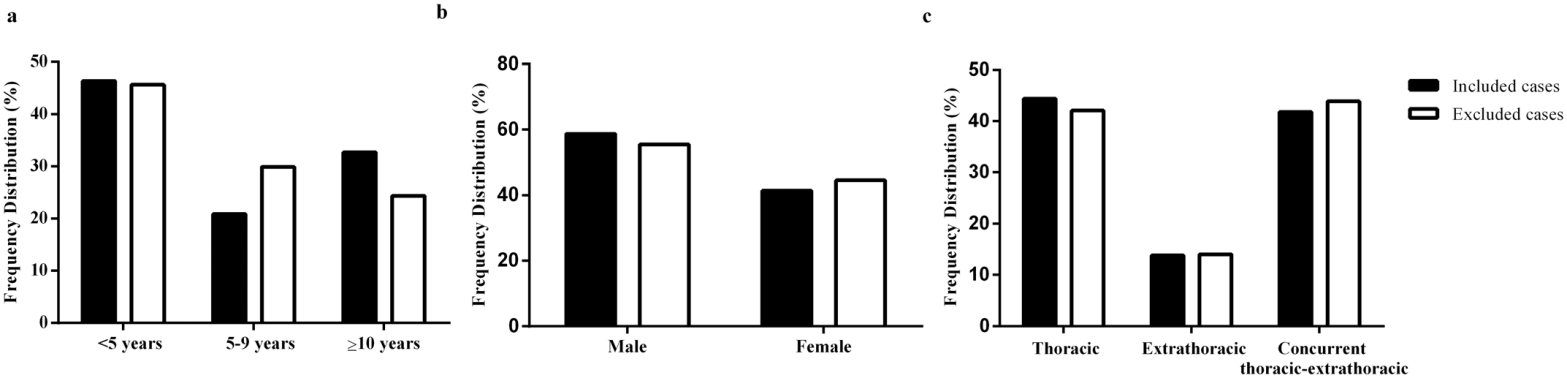
Comparison of frequency distribution of age (Panel a), sex (Panel b) and disease site (Panel c) between the 196 culture-confirmed cases diagnosed in the Children’s Hospital of Chongqing Medical University (CHCMU) during 2008–2013 that were included in the study sample and the 164 culture-confirmed cases diagnosed at CHCMU during the same time period that were not included in the study sample. Chi-square test showed that there was no significant differences in age, sex and disease site frequency distribution between the included and excluded cases (p>0.05).

Given that only 54.4% (196/360) of all the mycobacterial culture-confirmed cases diagnosed at CHCMU during the study period were included in the study, we assessed the potential selection bias of our study sample by comparing the distribution of age and sex, two most important determinants for clinical presentation of pediatric TB and disease site between the 196 study patients and the 164 culture-confirmed cases not included in the study. The comparison showed no significance between the study sample and excluded culture-confirmed cases (Fig 1), and the similar frequency distribution of age, sex and disease site minimizes the degree of concern for selection bias. Of the 196 study patients, 115 (58.7%) were male and 81 (41.3%) were female. The age of the study patients ranged between one month and 15 years, with a median age of 6.3 years. Children aged under five years accounted for 46.4% (91/196) of the study sample, among which 36.3% (62/196) aged under two years. Twenty-one percent (41/196) of the study patients were five to nine years old, and the remaining 32.7% (64/196) were older than 10 years. The majority (63.8%, 125/196) of the study patients were from rural areas. Exclusive thoracic TB cases accounted for 44.4% (87/196) of the study sample, while concurrent thoracic-extrathoracic TB and exclusive extrathoracic TB cases accounted for 41.8% (82/196), and 13.8% (27/196) respectively of the study sample. The study sample consisted of 98.5% (193/196) new cases and 1.5% (3/196) previously-treated cases. Previously-treated cases were defined as those who had been treated with anti-TB drugs for more than one month previously [3]. All the study patients were HIV sero-negative. Of the 175 patients for whom information about BCG vaccination was available, 71.4% (125/175) were vaccinated with BCG.

### Drug resistance pattern and frequency

**Fig 2:**
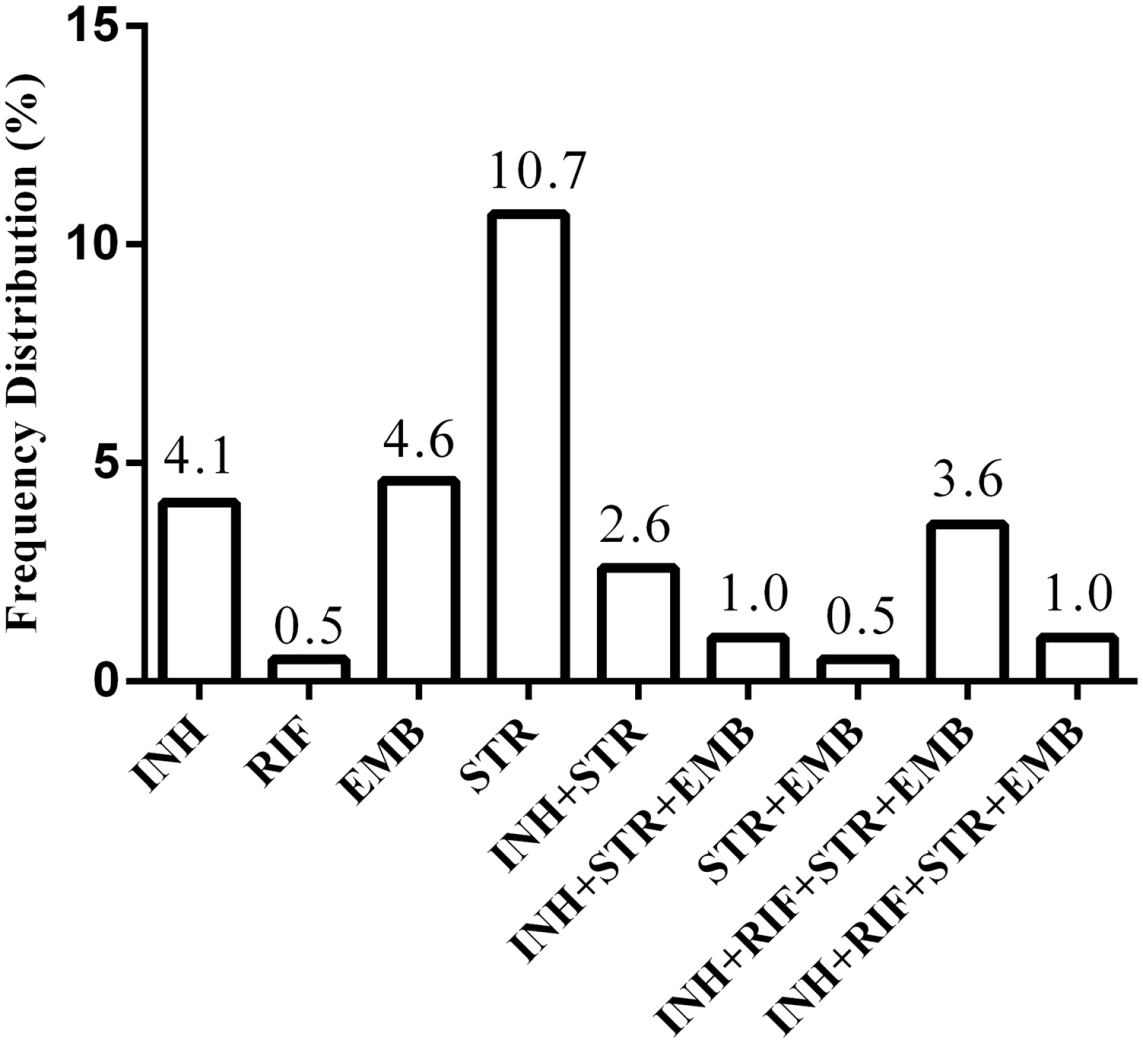
Frequency distribution of drug-resistant patterns observed among all 196 culture-confirmed tuberculosis patients diagnosed in the Children's Hospital of Chongqing Medical University during 2008–2013. INH-isoniazid, RIF-rifampicin, EMB-ethambutol, STR-streptomycin.

More than one quarter of the study patients (28.6%, 56/196) had an isolate that was resistant to at least one of the four first-line anti-TB drugs tested. Among the 56 DR-TB cases, nine different resistance patterns were observed (Fig 2). Mono-resistance to STR (10.7%, 21/196), a drug that has no longer been used in the standard anti-TB treatment regimens in many countries of the word, but that is still commonly used in China [18], was the most commonly observed DR pattern, while mono-resistance to EMB (4.6%, 9/196) and mono-resistance to INH (4.1%, 8/196) were, respectively, the second and the third most commonly observed (Fig 2). MDR-TB was found in 4.6% (9/196) of the study sample (Table 1). Of the nine MDR-TB cases, seven (77.8%) were resistant to all of the four first-line drugs. More importantly, five of these seven cases were from of the same area, Pengshui Miao and Tujia Autonomous County of Chongqing. In addition, resistance to INH or RIF (not to both), the ‘pro-type’ of MDR, accounted for 8.2% (16/196) of the study sample (Table 1). Of the three cases with previous anti-TB treatment, one was resistant to all of the four first-line drugs tested, and the remaining two were pansensitive. DR patterns of isolates from different anatomic sites of a single patient showed identical DR patterns.

**Table 1:** Drug susceptibility to first-line anti-tuberculosis drugs among 196 culture-confirmed pediatric tuberculosis cases diagnosed in the Children's Hospital of Chongqing Medical University during 2008–2013.

Susceptibility	No.	% (95% CI)
**Pansensitive to all four first-line drugs**	140	71.4 (65.1–77.7)
**R**^a^ **to any first-line drug**	56	28.6 (22.3–34.9)
**Isoniazid-R**	24	12.2 (7.6–16.8)
**Rifampin-R**	10	5.1 (2.0–8.2)
**Ethambutol-R**	19	9.7 (5.6–13.8)
**Streptomycin-R**	37	18.9 (13.4–24.4)
**Isoniazid or rifampin (not for both) -R**	16	8.2 (4.4–12.0)
**Multidrug resistance**	9	4.6 (1.7–7.5)

^a^ R represents resistant.

### Patient characteristics associated with DR-TB and MDR-TB

In this study, we did not find any statistically significant difference in the distribution of demographic characteristics (sex, age, residence area) between pansensitive and DR groups (Table 2), nor between pansensitive and MDR-TB groups (Table 3), or between non-MDR DR, and MDR groups (Table 4). However, the frequency distribution of clinical characteristics demonstrated some significant differences between each pair of the comparative groups mentioned above (Tables 2–4). Patients with a negative AFB smear were overrepresented in the DR group, compared with the pansensitive group; being AFB smear negative was independently associated with DR-TB (adjusted OR, 2.33; 95% CI, 1.13–4.80) (Table 2). The MDR-TB group contained a significantly higher proportion (88.9%) of cases with concurrent thoracic-extrathoracic involvement than the pansensitive group (40.0%) (Table 3) and non MDR DR-TB group (38.3%) (Table 4). In addition, concurrent thoracic-extrathoracic involvement was significantly associated with the MDR-TB (adjusted OR, 9.49; 95% CI, 1.05–85.92), compared to the pansensitive TB group (Table 3). Furthermore, statistically significant association was also found between concurrent thoracic-extrathoracic (adjusted OR, 19.46; 95% CI, 1.32–286.53) and MDR-TB when MDR-TB was compared to non MDR DR-TB cases (Table 4).

**Table 2:** Comparison of demographic and clinical characteristics between pansensitive (n = 140) and DR-TB^a^ (n = 56) diagnosed in the Children's Hospital of Chongqing Medical University during 2008–2013 using logistic regression models.

Characteristic	Pansensitive TB	DR-TB	p value^b^	DR-TB vs. Pansensitive TB	
	N (%)	N (%)		Crude OR (95% CI)	Adjust OR (95% CI)
**Sex**			0.36		
**Male**	85 (60.7)	30 (53.6)		Ref	Ref
**Female**	55 (39.3)	26 (46.4)		1.34 (0.72–2.50)	1.39 (0.70–2.77)
**Age**			0.80		
**<5 year**	67 (47.9)	24 (42.9)		Ref	Ref
**5–9 years**	29 (20.7)	12 (21.4)		1.16 (0.51–2.62)	1.25 (0.50–3.11)
**≥ 10 years**	44 (31.4)	20 (35.7)		1.27 (0.63–2.57)	1.74 (0.79–3.81)
**Residence**			0.67		
**Rural**	88 (62.9)	37 (66.1)		Ref	Ref
**Urban**	52 (37.1)	19 (33.9)		0.87 (0.45–1.67)	0.84 (0.41–1.73)
**BCG history**^c^			0.56		
**Yes**	87 (70.2)	38 (74.5)		Ref	Ref
**No**	37 (29.8)	13 (25.5)		0.80 (0.39–1.68)	0.68 (0.31–1.49)
**Disease site**			0.42		
**Thoracic**	62 (44.3)	25 (44.6)		Ref	Ref
**Extrathoracic**	22 (15.7)	5 (8.9)		0.56 (0.19–1.65)	0.61 (0.20–1.92)
**Concurrent thoracic-extrathoracic**	56 (40.0)	26 (46.4)		1.15 (0.60–2.22)	1.16 (0.55–2.42)
**AFB**^d^ **smear**^e^			0.03		
**Positive**	69 (50.4)	18 (32.7)		Ref	Ref
**Negative**	68 (49.6)	37 (67.3)		2.09 (1.08–4.02)	2.33 (1.13–4.80)

^a^ DR-TB represents drug-resistant tuberculosis.

^b^ Based on Chi-square test.

^c^ Based on 175 cases for which information on BCG vaccination history is available.

^d^ AFB represents acid-fast bacilli.

^e^ Based on 192 cases for which AFB smear was conducted.

**Table 3:** Comparison of demographic and clinical characteristics between pansensitive (n = 140) and MDR-TB^a^ (n = 9) diagnosed in the Children's Hospital of Chongqing Medical University during 2008–2013 (n = 149) using logistic regression models.

Characteristic	Pansensitive TB	MDR-TB	p value^b^	MDR-TB vs. Pansensitive TB	
	N (%)	N (%)		Crude OR (95% CI)	Adjust OR (95% CI)
**Sex**			0.74		
**Male**	85 (60.7)	5 (55.6)		Ref	Ref
**Female**	55 (39.3)	4 (44.4)		1.24(0.32–4.81)	1.74 (0.33–9.19)
**Age**			0.08		
**<5 year**	67 (47.9)	1 (11.1)		Ref	Ref
**5–9 years**	29 (20.7)	3 (33.3)		6.93 (0.69–69.46)	9.10 (0.68–121.54)
**≥ 10 years**	44 (31.4)	5 (55.6)		7.61 (0.86–67.38)	9.43 (0.92–96.16)
**Residence**			0.49		
**Rural**	88 (62.9)	7 (77.8)		Ref	Ref
**Urban**	52 (37.1)	2 (22.2)		0.49 (0.10–2.42)	0.42 (0.07–2.53)
**BCG history**^c^			0.70		
**Yes**	87 (70.2)	5 (62.5)		Ref	Ref
**No**	37 (29.8)	3 (37.5)		1.41 (0.32–6.21)	0.68 (0.12–3.84)
**Disease site**			0.02		
**Thoracic**	62 (44.3)	1 (11.1)		Ref	Ref
**Extrathoracic**	22 (15.7)	0 (0.00)		NA	NA
**Concurrent thoracic-extrathoracic**	56 (40.0)	8 (88.9)		8.86 (1.07–73.06)	9.49 (1.05–85.92)
**AFB**^d^ **smear**^e^			1.00		
**Positive**	69 (50.4)	5 (55.6)		Ref	Ref
**Negative**	68 (49.6)	4 (44.4)		0.81 (0.21–3.15)	0.68 (0.12–3.78)

^a^ MDR-TB represents multidrug-resistant tuberculosis.

^b^ Based on Fisher's exact test.

^c^ Based on 132 cases for which information on BCG vaccination history is available.

^d^ AFB represents acid-fast bacilli.

^e^ Based on 146 cases for which AFB smear was conducted.

**Table 4:** Comparison of demographic and clinical characteristics between non MDR^a^ DR-TB^b^ (n = 47) and MDR-TB (n = 9) among 56 DR-TB patients diagnosed in the Children's Hospital of Chongqing Medical University during 2008–2013 using logistic regression models.

Characteristic	Non MDR DR-TB	MDR-TB	p value^c^	MDR-TB vs. non MDR DR-TB	
	N (%)	N (%)		Crude OR (95% CI)	Adjust OR (95% CI)
**Sex**			1.00		
**Male**	25 (53.2)	5 (55.6)		Ref	Ref
**Female**	22 (46.8)	4 (44.4)		0.91 (0.22–3.82)	1.78 (0.18–17.94)
**Age**			0.08		
**<5 year**	23 (48.9)	1 (11.1)		Ref	Ref
**5–9 years**	9 (19.1)	3 (33.3)		7.67 (0.70–83.74)	13.34 (0.69–259.70)
**≥ 10 years**	15 (31.9)	5 (55.6)		7.67 (0.81–72.26)	12.50 (0.59–265.89)
**Residence**			0.70		
**Rural**	30 (63.8)	7 (77.8)		Ref	Ref
**Urban**	17 (36.2)	2 (22.2)		0.50 (0.09–2.71)	0.41 (0.04–4.82)
**BCG history**^d^			0.40		
**Yes**	33 (76.7)	5 (62.5)		Ref	Ref
**No**	10 (23.3)	3 (37.5)		1.98 (0.40–9.77)	1.13 (0.10–12.55)
**Disease site**			0.02		
**Thoracic**	24 (51.1)	1 (11.1)		Ref	Ref
**Extrathoracic**	5 (10.6)	0 (0.00)		NA	NA
**Concurrent thoracic-extrathoracic**	18 (38.3)	8 (88.9)		10.67 (1.22–93.13)	19.46 (1.32–286.53)
**AFB**^e^ **smear**^f^			0.14		
**Positive**	13 (28.3)	5 (55.6)		Ref	Ref
**Negative**	33 (71.7)	4 (44.4)		0.32 (0.07–1.36)	0.14 (0.02–1.19)

^a^ MDR represents multidrug-resistant.

^b^ DR-TB represents drug-resistant tuberculosis.

^c^ Based on Fisher's exact test.

^d^ Based on 51 cases for which information on BCG vaccination history is available.

^e^ AFB represents acid-fast bacilli.

^f^ Based on 55 cases for which AFB smear was conducted.

## Discussion

The wide spread of *M*. *tuberculosis* infection that is resistant to anti-TB drugs represents a major challenge to global TB control. This study was the first to address specifically the epidemiology and clinical characteristics of pediatric DR-TB in a high TB-burden area of China. The major findings of this study include: 1) More than one quarter (28.6%, 56/196) of the study patients exhibited resistance to at least one of the four first-line anti-TB drugs tested, with mono-resistance to STR (10.7%, 21/196), a commonly used first line anti-TB drug in pediatric TB treatment in China [18], being the most commonly observed DR pattern; 2) MDR-TB accounted for 4.6% (9/196) of the study sample, of which the majority (55.6%, 5/9) came from a single area, Pengshui Miao and Tujia Autonomous County, Chongqing; 3) A significant, independent association was found between being acid-fast bacilli-smear negative and DR-TB (adjusted OR, 2.33; 95% CI, 1.13–4.80) and between having concurrent thoracic-extrathoracic involvement and MDR-TB (adjusted OR, 9.49; 95% CI, 1.05–85.92), respectively.

Despite the increasing number of study reports of DR-TB in the literature, there are limited epidemiological reports on pediatric DR-TB. The present study found a significantly higher rate of DR-TB (28.6%) when compared to two similar hospital-based studies conducted in India (20.47%) and South Africa (15.5%) [19, 20], while much lower rate compared to the recent study in China (55%) [5]. The rate of MDR-TB among our study patients is close to that found in India (4.6% vs. 3.9%) but lower than that found in South Africa (4.6% vs. 8.8%) [19, 20] and the previous study in China (4.6% vs. 22%) [5]. The fact that the majority of the pediatric MDR-TB cases in both the present study (77.8%, 7/9) and in the South Africa study (84.6%, 11/13) were new cases suggests the important role of ongoing transmission in the occurrence of pediatric MDR-TB in high TB burden countries [20], like China and South Africa. It is also intriguing to note that the reported national rates of MDR-TB (5.7% among new TB cases and 25.6% among previous treated cases) in China is actually higher than that reported in South Africa (1.8% among new TB cases and 6.7% among previously-treated cases) [3, 21]. This may suggest that there is less transmission of MDR-TB from adult MDR-TB cases to children in China than in South Africa. It may also reflect a possible under detection of pediatric MDR-TB cases in the present study. Although both DR and MDR rates in the sample of the present study were slightly lower than those found among new TB cases in the last national DR-TB survey of China (28.6% vs. 34.2% for DR-TB and 4.6% vs. 5.7% for MDR-TB) [3], the present study revealed a serious epidemic of pediatric DR-TB in Chongqing, China. Both the current study and the previous pediatric DR-TB study in China found mono-resistance to STR as the most commonly observed resistance pattern [5], which is consistent with the last national DR-TB survey and suggests that investigation of pediatric DR-TB can provide some insight into the problem of DR-TB in the population at large [3]. Although STR was no longer used as a first-line anti-TB drug in pediatric TB treatment according to WHO’s guidelines [13], it is still commonly used for treatment pediatric TB in China [18]. In the recent years, the use of STR began to decrease in China due to its serious side effects such as ototoxicity among children. The high mono-STR resistance rate among pediatric isolates from China found by previous and present studies also indicate an urgent need for minimizing or eliminating the use of STR in pediatric TB treatment.

Another important finding regarding MDR-TB generated by the present study is that of the nine patients with MDR-TB in our study patients, five (55.6%) resided in Pengshui Miao and Tujia Autonomous County, Chongqing. Pengshui is located in a mountainous region that is 270 kilometers away from the city of Chongqing. Residents in Penshui are known to have limited access to health care due to inconvenient transportation and the low level of economic development in this area [22]. A previous report by Chongqing Tuberculosis Institute showed that Pengshui had an average annual TB incidence of 161.7 per 100,000 during 2009 to 2013 [23]. However, this previous study did not investigate the rates of DR-TB and MDR-TB. It has been noted that most MDR-TB cases in China resulted from primary transmission [3]. In order to control MDR-TB in China, the first step is to identify those hotspots for MDR-TB transmission. Studies on the regional transmission of MDR-TB are scarce in China. The finding that of the nine patients with MDR-TB in the present study, five (55.6%) came from the same social-economically underprivileged geographic area is alarming and suggests the existence of an ongoing MDR-TB transmission hotspots in this area. This finding also showed that studying pediatric TB, which has long been neglected in most national TB programs [24], is critical to the goal of national TB control program as it has significant potential to help in identifying transmission hotspots in a community.

The current study identified associations between two clinical characteristics and DR- and MDR-TB, respectively. The association between negative smear and DR-TB suggests that more attention should be given to smear negative pediatric TB in the management of pediatric TB. It also suggests that those smear-negative patients that often are not the primary targets for TB control programs might be the potential sources of DR-TB transmission given that a study conducted in San Francisco reported that patients with smear-negative culture-positive TB appeared responsible for about 17% of TB transmission [25].

In addition, the present study showed, for the first time, that having concurrent thoracic-extrathoracic involvement was an independent predictor for MDR-TB. This finding suggests that MDR *M*. *tuberculosis* isolates may have an enhanced ability to disseminate from the initial infection site and to cause disease in both the initial infection site within the thorax and the extrathoracic site that the bacteria disseminated to, compared to drug susceptible and non MDR-DR strains. Such enhanced ability to disseminate and to cause disease in multiple anatomic sites implies an elevated virulence with potential for devastating worldwide impact. This finding is important as previous studies reported that most antibiotic resistance mechanisms were associated with a fitness cost of the bacteria expressed as a reduced bacterial growth rate, and that such fitness cost can result in a reduction of the virulence of the DR organisms [7–10]. However, as mentioned earlier, studies on the fitness cost of DR and MDR *M*. *tuberculosis* strains in experimental animals and epidemiological studies of human TB diseases have generated different findings [7–10, 26]. Bacteria fitness depends on the interplay of a complex set of factors related to both the infected human host and the infecting pathogen that may not be able to be mimicked completely by experimental animal models. Therefore, epidemiological study of associations between TB patient clinical characteristics and DR- and MDR-TB are necessary to generate additional insight into the pathogenesis of DR and MDR *M*. *tuberculosis* infection in humans. We also note that the proportion of extrathoracic TB in our study sample is higher than those found in some of the previous studies conducted in Spain, South Africa, Nepal and Vienam [11, 27–29]. Similarly, another pediatric TB study in China reported a high percentage of extrathoracic TB (54.0%, 655/1212) [30]. The high percentage of extrathoracic TB may result from the prevalence of *M*. *tuberculosis* Beijing family strains among pediatric TB in China [31], because of its known association with extrathoracic TB [27].

This study has several limitations. First, the study sample involved *M*. *tuberculosis* isolates from only 54.4% (196/360) of the mycobacterial culture-confirmed cases and culture confirmed cases generally represent cases with more severe types of disease. However, DST can only be performed with mycobacterial isolates achieved from culture-confirmed patients, which is an inevitable limitation for DR-TB studies. In addition, the resistance to pyrazinamide, another commonly used first-line anti-TB drug was not included in the present study due to the high probability for generating false positive and inconsistent DST results as reported previously [32]. Finally, due to the extremely small sample size (3/196, 1.5%) of previously treated patients, the effect of having previous TB treatment on the risk for having DR- or MDR-TB was not assessed in the present study.

## Conclusions

To summarize, this study extends the knowledgebase of DR-TB in children and reveals an alarming situation of pediatric DR- and MDR-TB in China. The study also provides new insight into the pathogenesis of DR and MDR *M*. *tuberculosis* strains. Taking together, the findings of the study suggest an urgent need for incorporating the investigation of pediatric TB into the national TB programs in countries, like China where TB transmission is prevalent, and highlights the importance of studying childhood TB to the goal of global TB control.
